# Neuroprotective Effects of Fingolimod in a Cellular Model of Optic Neuritis

**DOI:** 10.3390/cells10112938

**Published:** 2021-10-28

**Authors:** Amritha A. Candadai, Fang Liu, Arti Verma, Mir S. Adil, Moaddey Alfarhan, Susan C. Fagan, Payaningal R. Somanath, S. Priya Narayanan

**Affiliations:** 1 Clinical and Experimental Therapeutics Program, College of Pharmacy, University of Georgia, Augusta, GA 30912, USA; amritha@uga.edu (A.A.C.); fliu1@augusta.edu (F.L.); Av01776@uga.edu (A.V.); madil@augusta.edu (M.S.A.); malfarhan@augusta.edu (M.A.); sfagan@augusta.edu (S.C.F.); sshenoy@augusta.edu (P.R.S.); 2 Charlie Norwood VA Medical Center, Augusta, GA 30912, USA; 3 Culver Vision Discovery Institute, Augusta University, Augusta, GA 30912, USA

**Keywords:** optic neuritis, multiple sclerosis, oxidative stress, neuroprotection, fingolimod

## Abstract

Visual dysfunction resulting from optic neuritis (ON) is one of the most common clinical manifestations of multiple sclerosis (MS), characterized by loss of retinal ganglion cells, thinning of the nerve fiber layer, and inflammation to the optic nerve. Current treatments available for ON or MS are only partially effective, specifically target the inflammatory phase, and have limited effects on long-term disability. Fingolimod (FTY) is an FDA-approved immunomodulatory agent for MS therapy. The objective of the current study was to evaluate the neuroprotective properties of FTY in the cellular model of ON-associated neuronal damage. R28 retinal neuronal cell damage was induced through treatment with tumor necrosis factor-α (TNFα). In our cell viability analysis, FTY treatment showed significantly reduced TNFα-induced neuronal death. Treatment with FTY attenuated the TNFα-induced changes in cell survival and cell stress signaling molecules. Furthermore, immunofluorescence studies performed using various markers indicated that FTY treatment protects the R28 cells against the TNFα-induced neurodegenerative changes by suppressing reactive oxygen species generation and promoting the expression of neuronal markers. In conclusion, our study suggests neuroprotective effects of FTY in an in vitro model of optic neuritis.

## 1. Introduction

Multiple sclerosis (MS) is an autoimmune disease of the central nervous system (CNS) prevalent in about 400,000 people in the US and 2.1 million people worldwide [1,2,3,4]. Approximately 20% of MS patients present with vision deficits associated with optic neuritis (ON) [5,6], and neurodegeneration characterized by loss of retinal ganglion cells, thinning of the nerve fiber layer, and axonal damage [7,8]. Parameters of visual function are utilized as necessary outcome measures in MS studies [9]. Although the current MS therapies target the inflammatory pathology, effects on the long-term neurodegenerative phases of the disease have not been shown. A treatment that effectively targets both aspects of MS would likely achieve preferred status as a disease-modifying agent.

Fingolimod (FTY720 or FTY), a sphingosine analog that functions as a potent immunosuppressive agent in the CNS, is approved for MS therapy, especially for highly active disease [10,11]. Once phosphorylated into its active form by the sphingosine-1-kinase, it acts as an agonist on sphingosine-1-phosphate (S1P) receptors [12,13,14,15] and induces internalization of S1P receptors after binding [16]. Pharmacologically FTY is known as an immunomodulatory drug. FTY exerts its immunosuppressive effect by lymphocyte sequestration and thus reduces the numbers of T and B cells in circulation [17,18]. FTY has been shown to exhibit neuroprotective properties in experimental models of Alzheimer’s, stroke, and Parkinson’s disease [19,20,21,22,23,24]. A recent study showed that FTY exerts neuroprotective and anti-inflammatory effects on the retina and optic nerve in a mouse model of MS, perhaps explaining the potent protective effects in patients [25]. However, the molecular mechanisms regulating the neuroprotective properties have not been studied in retinal neurons. The current study was performed to assess the neuroprotective potential of FTY in an in vitro model of optic neuritis.

The R28 rat neuro-retinal cell line treated with TNFα was standardized to mimic MS-mediated neuronal injury in vitro. The cellular model was chosen based on the studies by Seigel et al. demonstrating the activity and/or expression of neuronal markers at the mRNA, protein, and functional levels in response to various stimuli [26,27]. The expression of neuron-specific markers such as microtubule associated protein 2 (MAP2), Syntaxin, neuron-specific enolase [NSE], Nestin, and receptors for neurotransmitters such as dopamine, serotonin, acetylcholine, and glycine justify the use of these cells to study CNS function [28]. Utilizing the in vitro experimental model of optic neuritis standardized in our laboratory using the tumor necrosis factor-α (TNFα) as an insult, the current study investigated the neuroprotective properties of FTY in reducing the TNFα-induced injury in R28 cells.

## 2. Materials and Methods

### 2.1. Cell Culture

Immortalized R28 postnatal day 6 rat neuro-retinal cells (heterogeneous population of cells derived from the parent cell line) (Cat # E1A-NR.3, Kerafast, Inc., Boston, MA, USA) were maintained in low-glucose DMEM medium (Cat # SH30021.01, Hyclone, Logan, UT, USA) supplemented with 10% fetal calf serum (Cat # SH30073.02, Hyclone, Logan, UT, USA), 0.225% Sodium bicarbonate (Cat # S8761, Sigma, St. Louis, MO, USA), 1X MEM non-essential amino acids (Cat # 11140-050, GIBCO, Waltham, MA, USA), 1X MEM vitamins (Cat # 11120-052, GIBCO, Waltham, MA, USA), 0.5 mM l-glutamine (Cat # 25030-081, GIBCO, Waltham, MA, USA), and 50 µg gentamicin (Cat # 15750-060, GIBCO, Waltham, MA, USA). The cells were differentiated to a neuronal phenotype with the help of 25 µg/mL laminin (Cat # 11243217001, Sigma, St. Louis, MO, USA) and 250 mM modified cyclic adenosine monophosphate (pCPT-cAMP) (Cat # C3912, Sigma, St. Louis, MO, USA) treatments, according to the published methods [29,30]. All other chemicals were purchased from Fisher Scientific, Waltham, MA, USA, unless otherwise mentioned.

### 2.2. The In Vitro Model of Optic Neuritis

Dose-response experiments were conducted to standardize the in vitro treatment with TNFα (recombinant rat tumor necrosis factor-α) (Cat # 510-RT, R&D Systems, Minneapolis, MN, USA) to induce neuronal injury in R28 cells. On day 0, cells were differentiated on 6-well culture plates (24 h), as described above. Treatments with TNFα at doses of 5, 10, 25, 50 ng/mL were initiated on day 1, followed by a 24 h incubation period. Cell viability with various doses of TNFα was compared against the control group with no treatment that depicted average growth and differentiation.

### 2.3. Treatments with Fingolimod

Once the effective dose of TNFα was established, experiments were conducted to identify an appropriate treatment concentration of FTY (Cat # 11975, Cayman Chemicals, Ann Arbor, MI, USA). Cells were pre-treated with FTY at concentrations of 2.5, 5, 10, 25, 50, and 100 nM for a 1 h incubation period before the TNFα treatment. Cell viability differences were compared among control (no treatment), TNFα-treated group, and TNFα co-incubated with varying FTY concentrations. FTY treatment alone at higher doses of 100, 200, and 500 nM were performed to test its cytotoxicity.

### 2.4. Cell Viability

The degree of viability of R28 cells post-treatment with TNFα and/or FTY was determined using the Trypan blue method [31]. Cells were seeded on six well plates at a density of 0.5 × 10^6^ per well. Following treatments, they were trypsinized and collected in labeled tubes respective to their grouping. Equal volumes of a sample of cell suspension and trypan blue dye were thoroughly mixed using a micropipette, from which 10 µL was injected into a cell counting chamber (Cat # 02-671-55A, Fischer Scientific, Waltham, MA, USA) for manual counting. Trypan blue dye stains dead cells blue, and the number of viable cells in all four 16-squared tiles of the chamber were counted. This was repeated in triplicates for each cell suspension sample, and cell viability was plotted as the percentage with respect to 100% control. All graphs are represented as Mean ± SEM.

### 2.5. Western Blot Analysis

R28 cells seeded at a density of differentiated on six well plates and treated with TNFα and/or FTY as described earlier, resulting in four groups: Control (no treatment), TNFα, TNFα + FTY, and Control + FTY groups. Cells were homogenized, and the lysate was collected in RIPA buffer (Cat # 20-188, EMD Millipore, Burlington, MA, USA) containing protease (Cat # 78430, Fischer Scientific, Waltham, MA, USA) and phosphatase inhibitors (Cat # 78428, Fischer Scientific, Waltham, MA, USA). Protein estimation was performed using the Bradford’s protein assay kit (Cat # 5000201, Bio-Rad Laboratories, Hercules, CA, USA). Samples with an equal amount of protein were prepared by using 4X Laemmli buffer (Cat # 161-0747, Bio-Rad Laboratories, Hercules, CA, USA) containing β-mercaptoethanol (Cat # O3446I-100, Fischer Scientific, Waltham, MA, USA). Samples were separated on SDS-PAGE and transferred to nitrocellulose membranes (Cat # 1620112, Bio-Rad Laboratories, Hercules, CA, USA). Membranes were blocked in 5% milk (Cat # 1706404, Bio-Rad Laboratories, Hercules, CA, USA) in tris-buffered saline with tween-20 (TBS-T) and incubated with respective primary antibodies (Table A1) overnight at 4 °C. Membranes were washed with 1× TBS-T and incubated in appropriate secondary antibodies. Signals were detected using enhanced chemiluminescence (ECL) (Cat # 32106, Fischer Scientific, Waltham, MA, USA). NIH Image J software was utilized to conduct densitometric analysis, and the intensity measurements were normalized to loading control. Experiments were repeated for a minimum of three times.

### 2.6. Immunofluorescence Staining

Cells seeded at a density of 15,000 to 20,000 cells per well on 8 well glass chamber slides (Cat # 154941, Fischer Scientific, Waltham, MA, USA) were treated according to the study design described previously. Following the 24 h incubation period, the culture media was removed, cells were washed with 1× PBS and fixed with 2% paraformaldehyde for 10 min. This was followed by a wash with PBS, and the chamber slides were stored in humidified containers at 4 ºC. Slides were brought to room temperature and washed with PBS before initiating the staining protocol. Permeabilization was achieved using 0.1% Triton X-100 in PBS for 5 min, followed by a PBS wash, and blocking with 10% donkey serum at room temperature for 1 h. Cells were washed and incubated with respective primary antibodies (Table A1) overnight. The next day, the cells were incubated with appropriate secondary antibodies for 2 h. The Slides were washed, and the chambers were separated from the glass slide. Cells were covered with a coverslip using a mounting medium containing DAPI and stored in 4 °C. Images were taken using a confocal microscope (LSM 780; Carl Zeiss, Thornwood, NY, USA) available at the Augusta University imaging core facility. For the Tuj1 and NSE quantitative analysis, similar thresholds were set for all the images. Three to five regions of interest (ROI) were randomly selected per chamber slide images, and the fluorescent intensity of immunoreactivity was measured (Integrated Density) using NIH Image J software. Along with the average fluorescence intensities of a given ROI, the average fluorescence intensity of areas without staining (background) was measured as well. The Corrected Fluorescence was calculated as Integrated Density—(Area of selected cells X Mean fluorescence of background readings). The values were then normalized relative to percentage of control group. Experiments were repeated a minimum of three times and details are provided under figure legends.

### 2.7. Cellular ROS Formation Using DCF Assay

CM-H2DCFDA (General Oxidative Stress Indicator, Thermofisher Scientific, cat# C6827) was used to measure reactive oxygen species (ROS) formation in TNFα treated cells and any changes in response to FTY treatment, as per the manufacturer’s instructions. Briefly, cells were washed with ice-cold PBS (three times), and then incubated with 10 µm CM-H2DCFDA (working solution) at 37 °C for 30 min under dark. Washed with ice-cold PBS (three times), cover-slipped using the mounting medium with DAPI stain (Vector laboratories), and the images were immediately taken by confocal microscope (LSM 780; Carl Zeiss, Thornwood, NY, USA). NIH Image J software was used for the analysis of fluorescent intensity. For single-cell quantification, single-cell was delineated and sampled at 40× from a random start point. Only cells with precise neuronal shape and specific nuclear staining with DAPI were analyzed. Three to five chamber slides per treatment were examined and the experiments were repeated three times.

### 2.8. Mitochondrial ROS Measurement

The production of superoxide by mitochondria in response to TNFα treatment and the impact of FTY on the mitochondrial ROS generation was measured using the MitoSOX™ Red reagent (Thermo Fisher Scientific, cat# M36008), following the manufacturer’s instructions. Briefly, cells on chamber slides were washed with ice-cold PBS (three times), incubated with 5 μM MitoSOX™ reagent working solution, and incubated for 10 min at 37 °C, protected from light. Chamber slides were then carefully washed with ice-cold PBS (three times), cover slipped, and images were immediately taken by confocal microscope (LSM 780; Carl Zeiss, Thornwood, NY, USA). NIH Image J software was used for the analysis of fluorescent intensity. Three to five chamber slides per treatment were examined and the experiments were repeated three times.

### 2.9. H_2_O_2_ Treatment and LDH Assay

Oxidative stress was induced on differentiated R28 cells using H_2_O_2_ (SigMA, USA) following the method of Song et al. [32] with some minor modifications. Briefly, R28 cells were treated with multiple concentrations of H_2_O_2_ (0.0, 0.2, 0.4, 0.6, 0.8, 1.0, and 1.5 mM) for 24 h and cellular cytotoxicity was measured. In order to determine the effect of FTY on H_2_O_2_-induced oxidative stress, cells were pre-treated with 25 nM FTY (as described previously) and changes in cytotoxicity was measured.

Lactate dehydrogenase (LDH) assay was used to determine cellular cytotoxicity. LDH released into the culture media from damaged cells was measured following the manufacturer’s instructions (CytoTox 96 non-radioactive cytotoxicity assay kit; Promega Corporation, Madison, WI, USA). The level of LDH release was normalized to the total LDH content following cell lysis in a medium. The absorbance was determined at 490 nm using a Multimode Microplate Reader (Berthold Technologies, Bad Wildbad, Germany). LDH release was expressed as a percentage of the maximum LDH released after cell lysis.

### 2.10. Statistical Analysis

All statistical analyses were performed with GraphPad Prism 7 (GraphPad Software Inc., La Jolla, CA, USA). Student t-test or Two-way ANOVA followed by Tukey’s multiple comparisons test was employed to analyze the groups. A *p* value less than 0.05 was considered as statistically significant. Results are presented as Mean ± SEM.

## 3. Results

### 3.1. Fingolimod Treatment Reduces TNFα-Induced Neuronal Injury

**Figure 1:**
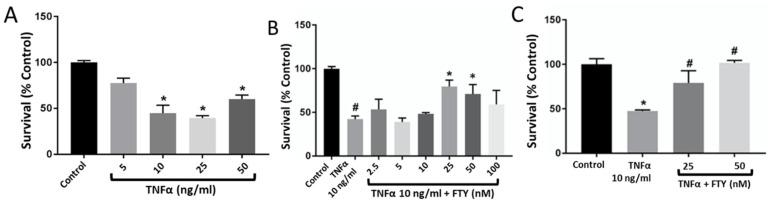
Dose-response effect of TNFα treatment on R28 cell survival and its reversal by co-treatment with fingolimod. (**A**) Neuronal damage was induced by treating R28 cells with various doses of TNFα for 24 h assessed by Trypan blue method. TNFα at 10ng/mL showed a marked reduction in cell survival and was chosen as the effective dose for further analysis. [*n* = 3; * *p* < 0.05 vs. dose 0]. (**B**) Cells were pre-treated with different doses of FTY, followed by TNFα (10 ng/mL), and cell survival was assessed at 24 h. Bar graph showing the effect of 25, 50, and 100 nM FTY on improving the rate of R28 cell survival [# *p* < 0.005 vs. Con; * *p* < 0.05 vs. TNFα]. (**C**) Bar graph indicating 25 nM as the optimal dose of FTY in protecting R28 cells from TNFα-induced injury in vitro [*n* = 3; * *p* < 0.001 vs. Con; # *p* < 0.01 vs. TNFα].

**Figure 2:**
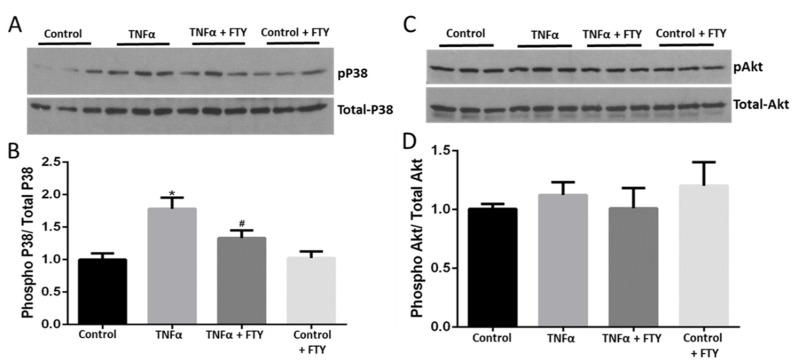
Fingolimod co-treatment mitigates TNFα-induced activation of cellular stress signaling. (**A**) Western blot images showing upregulation of phospho-p38 MAPK levels in response to TNFα (10 ng/mL) treatment in R28 cells, which was reduced in the presence of FTY. (**B**) Bar graph showing band densitometry quantification of Western blots indicating the effects of FTY in suppressing P38 MAPK activation induced by TNFα treatment [*n* = 6; * *p* < 0.05 vs. Con; # *p* < 0.05 vs. TNFα]. (**C**) Western blot images showing no changes in phospho-Akt (Ser473) levels in response to either TNFα (10 ng/mL) and/or FTY in R28 cells. (**D**) Bar graph showing band densitometry quantification of Western blots indicating the no changes in phospho-Akt (Ser473) levels in response to either TNFα (10 ng/mL) and/or FTY in R28 cells [*n* = 6; NS vs. TNFα].

We found that TNFα treatment resulted in a significant reduction (*p* < 0.05) in cell viability at doses of 10 ng/mL (44.6 ± 24.8%), 25 ng/mL (39.8 ± 8.7%), and 50 ng/mL (60.2 ± 13.0%) compared to the untreated control (Figure 1A). Our findings suggested that TNFα at 10 ng/mL desirably reduced the percentage of viable cells by nearly half that of the control group. In the next step, cells were pre-treated with fingolimod at concentrations of 0, 2.5, 5, 10, 25, 50, and 100 nM for 1 h prior to TNFα induction (10 ng/mL). Our results showed that FTY concentrations at 25 nM (79.7 ± 17.7%) and 50 nM (71.0 ± 32.3%) significantly prevented the TNFα-induced injury (*p* < 0.001) (Figure 2B). Experiments with high-dose FTY treatment alone resulted in cytotoxicity at doses above 100 nM. Doses of 100, 200, and 500 nM showed a decreasing viable cell count of 14.5 × 10^4^, 3.5 × 10^4^, and 2 × 10^4^, respectively, compared to an average viable cell count of 47.5 × 10^4^ in control cells without FTY treatment (not shown). Based on our findings, 25 nM was chosen as the dose of FTY to be used in further studies (Figure 1C).

### 3.2. Fingolimod Attenuates Cellular Stress and Survival Signaling

Changes in phosphorylated p38 MAP kinase expression were assessed to characterize cellular stress by Western blotting. Figure 2A shows that TNFα augmented the level of p-P38 MAPK, and this increase was markedly prevented in the presence of FTY. Moreover, we found that FTY treatment alone did not affect levels of p-P38 MAPK. Our quantification data demonstrated that in the presence of FTY, levels of p-P38/total-P38 were significantly reduced (*p* < 0.05) versus the TNFα group (Figure 2B). Changes in phosphorylated Akt levels were tested to assess cell survival using Western analysis. Figure 2C shows no changes in the expression of p-AKT with TNFα induction. Consistently, our quantification data also showed no significant decrease in levels of p-Akt/t-Akt in the presence of TNFα versus control (Figure 2D).

### 3.3. Effect of Fingolimod on Neuronal Cell Death

**Figure 3:**
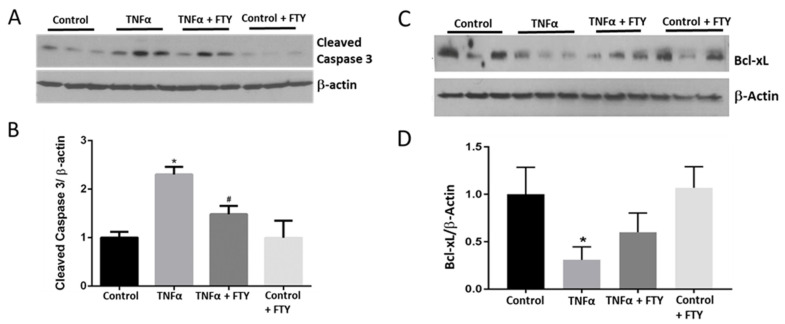
Fingolimod co-treatment blunted TNFα-induced activation of cleaved caspase-3 and expression of Bcl-xL. (**A**) Representative Western blot data showing increased expression of cleaved caspase-3 with TNFα treatment, which was reduced in the presence of FTY. (**B**) Bar graph showing band densitometry quantification indicating increased cleaved caspase-3 expression with TNFα treatment [* *p* < 0.05 vs. Con; *n* = 3] and its reversal by co-treatment with FTY [* *p* < 0.005 vs. control; # *p* < 0.05 vs. TNFα; *n* = 4]. (**C**) Western blot images showing reduced expression of Bcl-xL with TNFα treatment, which was increased in the presence of FTY. (**D**) Bar graph showing band densitometry quantification of Western blots indicating reduced Bcl-xL expression with TNFα treatment [* *p* < 0.05, *n* = 3]. The changes observed in response to FTY, however, were not statistically significant.

To evaluate apoptotic changes, Western blot analyses using apoptotic marker cleaved caspase-3, and anti-apoptotic marker Bcl-xL were performed. An upregulated expression on cleaved caspase-3 along with a decreased level of Bcl-xL in the presence of TNFα was observed. In the co-treatment group with TNFα and FTY, we observed a reversal in these changes (Figure 3). Quantification data showed a significant increase in the levels of cleaved caspase-3 (*p* < 0.01) versus control, while FTY treatment significantly reversed this effect (*p* < 0.05) versus the TNFα group (Figure 3B). TNFα caused a significant decrease in levels of Bcl-xL (*p* < 0.05) compared to the control group. However, the difference observed in response to FTY co-treatment was not statistically significant compared to the TNFα group (Figure 3D).

### 3.4. Effect of Fingolimod on TNFα-Induced Neuronal Damage

**Figure 4:**
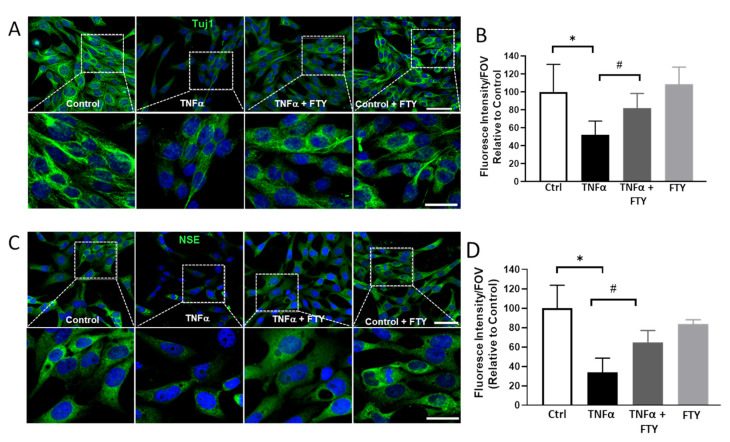
Fingolimod depicts protection against neuronal damage evidenced by immunofluorescence staining of marker proteins. (**A**) Representative confocal images showing the impact of TNFα treatment on neurofilament, Tuj1 (β-tubulin class III) indicating neurodegeneration, which was reduced by co-treatment with FTY. High magnification images of the boxed areas, indicating reduced Tuj1 expression, are presented in the lower panel. Scale bar 50 µm. (**B**) Bar graph showing the quantification of Tuj1 level in response to TNFα treatment, and the protective effect by co-treatment with FTY (**C**) Representative confocal images showing the changes in neuronal enolase (NSE) in response to TNFα and FTY treatments. Lower panel show high magnification images of the boxed areas, indicating reduced levels in TNFα treated group and the improved NSE expression in response to FTY co-treatment. Scale bar 50 µm. (**D**) Bar graph showing the quantification of Tuj1 and NSE levels in response to TNFα treatment, and the effect by co-treatment with FTY [* *p* < 0.01 vs. control; # *p* < 0.01 vs. TNFα, *n* = 3 per group].

Immunofluorescence studies were conducted to study the neurodegenerative changes observed in R28 cells in response to the different treatments. β-tubulin III, also called Tuj1 contributes to microtubule formation in neuronal cell bodies and axons and plays important roles in axonal transport and cell differentiation. It is a useful marker for the detection of injury-related alterations [33]. Neuron specific enolase (NSE) is widely used and accepted as a neuronal marker, and is expressed by mature neurons and cells of neuronal origin [34]. In the present study, immunostaining with Tuj1 and NSE revealed the degenerative changes induced by TNFα, which was attenuated with FTY treatment (Figure 4A,C). Tuj1 expression was downregulated in TNFα-treated cells; however, we observed that FTY was able to protect the cells against neurofilament damage (Figure 4A lower panels). Consistently, the NSE marker was found to be reduced in the presence of TNFα, and the reduction was prevented by FTY treatment (Figure 4B,D).

### 3.5. Effect of Fingolimod on ROS Formation

**Figure 5:**
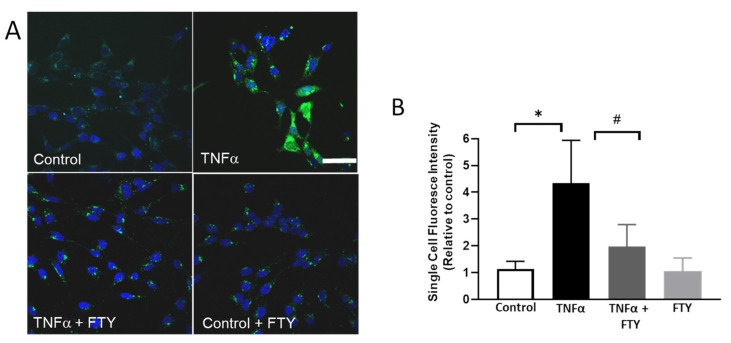
Fingolimod reduced TNFα-induced ROS formation. (**A**) Representative images showing the impact of FTY on reactive oxygen species (ROS) formation. H2DCFDA (DCF) assay was used to assess the generation of ROS. Scale bar 50 µm. (**B**) Quantification of single cell fluorescence intensity showing of the increased ROS formation in response to TNFα treatment, which was reduced in the presence of FTY [* *p* < 0.01 vs. control; # *p* < 0.01 vs. TNFα, n = 3 per group].

Changes in ROS formation were studied using DCF assay. As shown in Figure 5A, ROS levels were observed to be markedly elevated in the neuronal cells in response to TNFα treatment. However, treatment with FTY downregulated the TNFα induced ROS generation. Our quantification studies demonstrate that the ROS levels are significantly higher in the TNFα group compared to control and are significantly reduced in response to FTY co-treatment (Figure 5B).

### 3.6. Effect of Fingolimod on Mitochondrial Dynamics and ROS Formation

**Figure 6:**
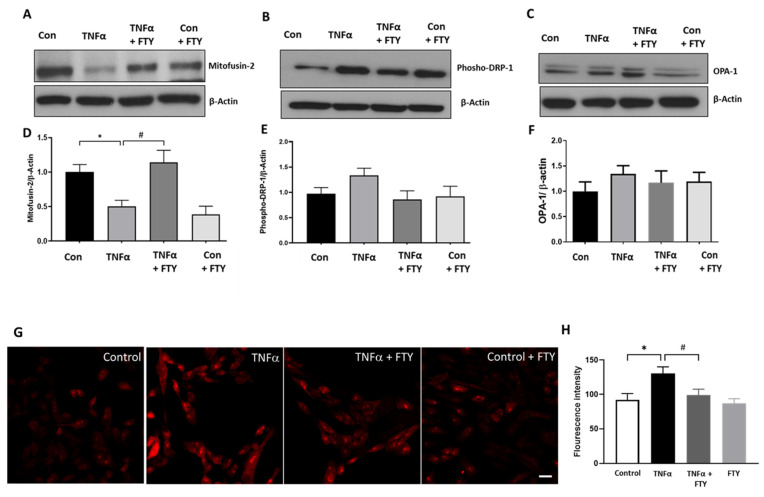
Changes in mitochondrial protein dynamics and ROS formation in response to Fingolimod treatment. (**A**–**C**) Representative Western blot data showing changes in expression of proteins associated with mitochondrial dynamics with TNFα treatment, and the effect of FTY cotreatment. (**D**–**F**) Respective bar graphs showing quantification of these proteins expression changes with TNFα treatment and any changes by co-treatment with FTY [* *p* < 0.05 vs. control; # *p* < 0.05 vs. TNFα; N = 3]. (**G**) Representative images of Mitosox Red staining showing the impact of TNFα on mitochondrial reactive oxygen species (ROS) formation and the effect of FTY on the treatment. Scale bar 20 µm. (**H**) Quantification of fluorescence intensity showing of the increased mitochondrial ROS formation in response to TNFα treatment, which was reduced in the presence of FTY [* *p* < 0.01 vs. control; # *p* < 0.01 vs. TNFα, *n* = 3 per group].

In the present study, we investigated the effect of FTY treatment on the changes in proteins related to mitochondrial dynamics, including DRP-1 (dynamin related protein-1), Mitofusin 2 and OPA-1 (optic atrophy 1). As illustrated in Figure 6A,D, expression of Mitofusin 2 was significantly reduced in TNFα treated R28 cells, while FTY treatment normalized the level of Mitofusin 2 in TNFα treated cells, similar to control levels. An increase in the level of p-DRP1 was observed in TNFα treated group, while treatment with FTY reversed this effect. However, these changes were not statistically significant. No marked differences were seen in the other mitochondrial proteins studied. Further, we investigated the impact of FTY treatment on mitochondrial ROS formation using MitoSox assay (Figure 6G,H). TNFα treatment resulted in the generation of mitochondrial ROS as evidenced by elevated fluorescence indicator. FTY treatment markedly reduced the level of mitochondrial ROS formed in response to TNFα treatment. Quantification results demonstrate that the mitochondrial ROS level is significantly upregulated in TNFα treated group and the treatment with FTY significantly reduced the effect (Figure 6H).

### 3.7. Fingolimod Attenuates H_2_O_2_-Induced Cellular Damage and Stress Signaling in R28 Cells

**Figure 7:**
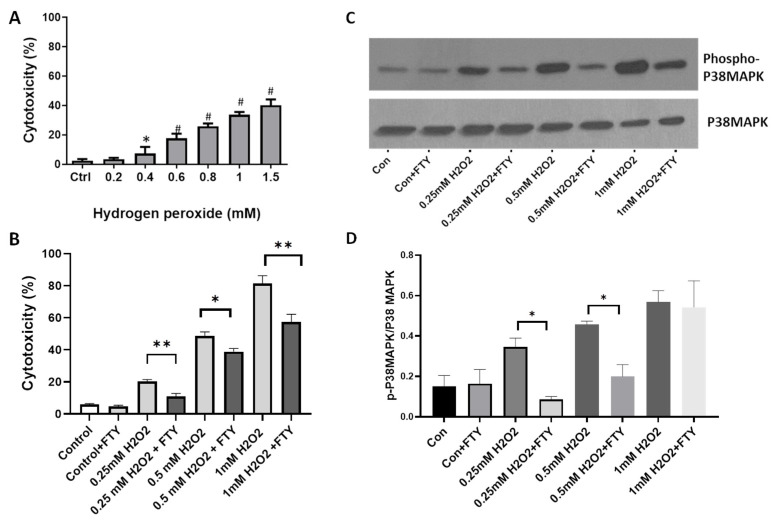
Fingolimod attenuates H_2_O_2_-induced cellular damage and stress signaling in R28 cells. (**A**) LDH assay results showing the dose-dependent effect of H_2_O_2_ treatment on R28 cells. * *p* < 0.05 vs control; # *p* < 0.01 vs. control and *n* = 3. (**B**) LDH assay showing the cytotoxic effect of H_2_O_2_ at selected concentrations on R28 cells and the protective effect of FTY on the treatments. * *p* < 0.05; ** *p* < 0.01. (**C**) Representative Western blot data showing changes in p-P38 MAPK with H2O2 treatment, and the protective effect of FTY co-treatment on differentiated R28 cells. (**D**) Bar graph showing quantification of p-P38MAPK proteins expression changes with H_2_O_2_ and FTY treatments. * *p* < 0.01 and the experiment was repeated three times.

To further assess the neuroprotective effect of FTY, experiments were performed using H_2_O_2_, another cellular stressor. Our results show that H_2_O_2_ induces cytotoxicity in differentiated R28 cells in a dose-dependent manner (Figure 7A). Treatment with FTY significantly reduced the cytotoxicity induced by TNFα at the various concentrations studied (Figure 7B). Our results indicate that H_2_O_2_ treatments significantly elevated p-P38 levels at all the concentrations studied, while FTY treatment significantly reduced the effect at two different contentions of H_2_O_2._ FTY treatment did not offer protection at a higher concentration, 1mM of H_2_O_2_ (Figure 7C).

## 4. Discussion

The present study was conducted to assess the potential neuroprotective action of FTY in an in vitro model of optic neuritis. Lack of effective treatment strategies to reduce neurodegeneration continues to be a major problem in the field of MS research. It is vital to understand the underlying mechanisms of MS-induced neuronal damage and dysfunction. Even though MS research on the pathophysiology and associated molecular mechanisms have evolved over decades of research, including our laboratory [35,36], the field lacks reliable in vitro models to study neurodegeneration. Synthetic molecules such as trimethyltin [37], oxaliplatin [38], and cuprizone [39], although successful in creating a neurodegenerative environment, do not accurately represent the neuroinflammatory changes observed in an MS brain. In MS, inflammatory leukocytes are believed to infiltrate the CNS to mediate demyelination and neuronal degeneration via cytokines upon activation of T lymphocytes and antigen-presenting cells (APCs) [40]. tumor necrosis factor-α (TNFα) is one of the primary cytokines that are present in elevated levels in active MS lesions, serum, and cerebrospinal fluid of MS patients [41]. Studies conducted on BV-2 microglial cell lines [42] and primary mixed neuronal and glial cultures [43] show the effect of TNFα-induced damage and apoptosis. Hence, utilizing the R28 neuro-retinal cells, we standardized an in vitro experimental model of neurodegeneration to assess the impact of fingolimod on TNFα-induced neuronal damage. Using a set of functional and biochemical analyses, our study demonstrates the neuroprotective properties of fingolimod in MS-associated optic neuritis in vitro.

We first demonstrated the dose-dependent effects of TNFα on neuronal cell viability in R28 neuro-retinal cells in vitro and identified the optimal dose of TNFα for further molecular characterization. The R28 cells are immortalized, rat retinal origin, heterogenous, precursor cells with differentiation potential [26]. According to the published methods, R28 cells were differentiated to neuronal phenotype with the addition of a modified form of cAMP and laminin and grown using DMEM [44,45,46]. Studies by Seigel et al. utilizing R28 cells demonstrate the expression of neuron-specific markers such as MAP2, Syntaxin, NSE, and Nestin, along with neurotransmission receptors [26,28]. Our study characterized the expression of neurofilament marker Tuj1, along with NSE in these cells, thus demonstrating the reliability of this model in evaluating the impact of FTY on the neuronal injury. In MS, one of the major clinical presentations observed in patients is optic neuritis [47]. Studies have shown that approximately 20% of patients present with inflammation of the optic nerve as their first symptom of MS [5,6]. Another study conducted by the North American Research Committee on multiple sclerosis (NARCOMS) showed that of the 9107 patients participating in the study, 60% reported signs of vision impairment, and 14% of these depicted moderate/severe/very severe impairment of vision [9]. Based on the available research, visual dysfunction is a common component of MS disease progression and an important determinant of quality of life.

Current MS therapies function by suppressing the inflammatory pathways and have unknown impact on the long-term neuronal damage, causing a major knowledge gap and emphasizes the need to identify a neuroprotective therapeutic agent. Therefore, our study focused on assessing the neuroprotective effect of FTY in an in vitro model of MS-induced optic neuritis. Fingolimod, a sphingosine-1-phosphate (S1P) receptor modulator, has previously been shown to prevent neurodegenerative mechanisms targeting an inflammatory CNS state in vitro, in vivo, and clinical settings, as detailed below. Studies on Parkinson’s disease models have shown a positive impact with FTY [19,20,21]. Mechanistically, it was found that the protective effects of FTY in Parkinson’s disease were correlated with the activation of survival pathway mediated by Akt/ERK1/2 and increased expression of a neuron-specific brain-derived neurotrophic factor (BDNF). In a model of Alzheimer’s, FTY was able to reverse the effect of damage by modulating the levels of different markers such as the Glial fibrillary acidic protein (astrogliosis marker), taurine (anti-inflammatory marker), and neuronal markers such as the N-acetyl aspartate and glutamate [23]. A meta-analysis was conducted by Liu et al. which included nine studies that focused on quantification of infarct volume and neurological deficit scoring in a transient middle cerebral artery occlusion model of ischemic stroke challenged with FTY [24]. The study concluded that FTY could be a possible candidate for stroke due to its protective effects on neurological deficit and infarct volume in eight of the nine included studies. Promising outcomes with FTY represent the need for further investigation to confirm the theories on its action as a neuroprotective agent. Utilizing the EAE (Experimental Autoimmune Encephalitis) model of MS, a recent study by Yang et al. investigated whether FTY is beneficial to the visual system [25]. Their results showed that FTY treatment offered neuroprotective and anti-inflammatory effects on the retina and optic nerve. FTY treatment alleviated EAE-induced gliosis, inflammation and reduced the apoptosis of RGCs and oligodendrocytes.

In response to FTY treatment, our studies found a reduction in the phosphorylation of p38 MAP kinase (a cellular stress signaling pathway) in TNFα-treated R28 cells. However, fingolimod treatment did not induce any changes in the levels of phosphorylated Akt, indicating its effect on cell survival is independent of Akt, with the conditions studied. TNFα-induced cell death was confirmed by the upregulation of cleaved caspase-3 (a cell death marker) expression along with reduced levels of Bcl-xL (an anti-apoptotic protein). These changes were reversed in response to FTY treatment, supporting its neuroprotective and anti-apoptotic function. FTY treatment also protected the retinal neurons against the TNFα-induced neuronal damage determined by the expression of neuronal markers.

Oxidative stress plays a critical part in the pathogenesis of various neurodegenerative diseases and neuroinflammation. There exists an increasing amount of data indicating that oxidative stress plays a major role in the pathogenesis of MS and optic neuritis [48]. Results from preclinical studies show that suppression of oxidative stress is a promising strategy for optic neuritis [49,50,51,52,53]. In the present study, we show that FTY mediated neuroprotection of R28 cells involves the regulation of ROS formation. A recent study performed on serum samples from patients with relapsing-remitting MS and healthy controls demonstrated that TFY treatment increased total antioxidant capacity [54]. Furthermore, FTY treatment reduced oxidative stress in experimental models of cardiomyopathy [55], multiple system atrophy [56], autism [57], and vitamin K-induced neurotoxicity [20].

Oxidative damages along with mitochondrial dysfunction are common characteristics of neurodegenerative diseases. Mitochondrial dysfunction is increasingly recognized as a major mechanism of MS associated pathologies [58,59,60]. The present study investigated the impact of FTY treatment on the changes in protein levels related to mitochondrial dynamics. Alterations in mitochondrial dynamics affect mitochondrial size and shape and impact mitochondrial metabolism and cell death. These events are controlled by mitochondrial dynamin-related GTPases, including mitofusin-1, mitofusin-2, OPA1, and DRP1. Our results show that one possible mechanism by which FTY offers neuroprotection of R28 neuronal cells via regulation of mitochondrial fusion. Altered levels of mitochondrial proteins were reported in retinal neurons in models of diabetic retinopathy [61] and glaucoma [62]. Results from our study are consistent with studies on neuroprotective properties of FTY in other models where FTY improved mitochondrial stability and restored mitochondrial dynamics under oxidative stress conditions [20,63,64,65]. Our present study did not investigate the changes in mitochondrial function.

Our study showed that FTY treatment reduces TNFα induced mitochondrial ROS formation in R28 cells. Other studies recently reported that FTY reduced mitochondrial dysfunction in a rat model of chronic cerebral hypoperfusion, reduced neuroinflammation and restored mitochondrial function in a model of Multiple System Atrophy [65], prevented mitochondrial dysfunction and protected neurons in prion protein-disease model [66]. Furthermore, we investigated the neuroprotective effect of FTY on R28 cells treated with H_2_O_2_, a cellular stressor known to induce oxidative stress. Our results reveal that FTY offers neuroprotection in response to oxidative stress-induced cellular damage. While FTY treatment rescued R28 cells at all concentrations of H_2_O_2_ studied, FTY did not significantly reduce phospho-P38 levels at higher concentration of H_2_O_2_, suggesting the possibility of other signaling pathways involved. Another possibility is that FTY at a different dose/time could reduce the phospho-P38 levels at higher concentrations of H_2_O_2_. Overall, these results indicate that FTY mediated neuroprotection could be offered through multiple mechanisms, including P38MAPK signaling. However, further studies are needed to confirm this observation.

**Figure 8:**
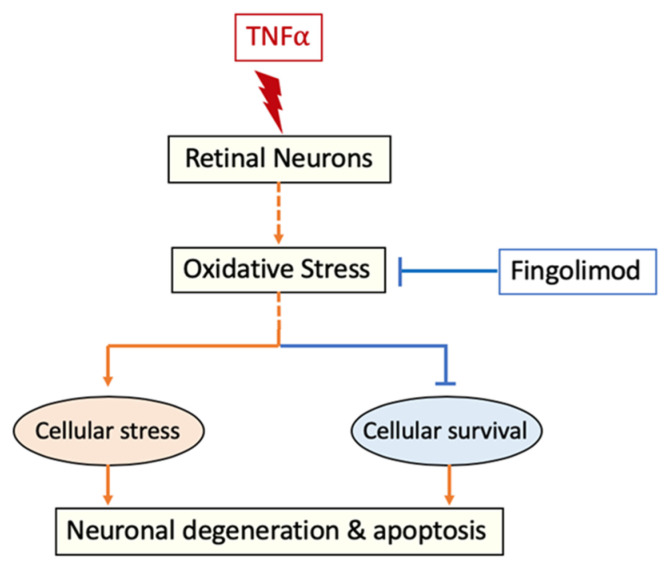
Proposed mechanism of FTY mediated neuroprotection. It is postulated that TNFα treatment induces cellular stress and cell death in retinal neurons by elevating oxidative stress. Treatment with FTY reduces TNFα-induced oxidative stress and improved neuronal survival.

Evidence as a regulator of oxidative stress, along with its immunomodulatory function, offers significant therapeutic potential to FTY in neuroinflammatory diseases such as optic neuritis. Figure 8 depicts the possible mechanisms of FTY mediated neuroprotection in response to TNFα-induced damage. Further studies are needed to define whether the effect of FTY on ROS level is direct or indirect and to delineate the molecular mechanisms of FTY mediated neuroprotection.

One limitation of our study is that it did not elucidate the role of cell survival pathways that are directly associated with TNFα and FTY action. The concentration of FTY used in our study (25 nM) corresponds to 7.6875 ng/mL. However, as per the manufacturer’s (Gilenya^®^) package insert, the concentration of active fingolimod phosphate in adult MS patients is 1.35 ng/mL. Lower concentrations used in our study, did not offer any neuroprotective effect. It is likely that the neurovascular unit could respond differently in response to other cytokines or injury mediators. This difference in human systemic concentration versus in vitro concentration in our models is an interesting aspect of the study and suggests that repurposing may be needed for the neuroprotective action of FTY in MS patients. Another limitation of the current study is that it is purely performed in a cellular model in vitro, which is not a true reflection of what happens in a complex in vivo set up. Nevertheless, the study provides reasonable optimism on the potential therapeutic benefits of FTY to treat ON. Studies performed on EAE model by Yang el tal demonstrated neuroprotective and anti-inflammatory effects on the retina and optic nerve, with no direct negative effects at the two different doses of FTY (0.3 and 1.0 mg/kg) utilized [25]. Results from our study complements the findings of Yang et al., where stronger neuroprotective effects on the visual system.

## 5. Conclusions

Overall, our study investigated the potential neuroprotective effects of FTY in an in vitro model of neurodegeneration. The R28 neuro-retinal cells are characterized as a successful platform for evaluating neuronal damage in the presence of TNFα, and its suppression with FTY. Furthermore, our studies demonstrated the antioxidant properties of FTY, a possible mechanism of neuroprotection. However, further studies are required to confirm the results. Based on the cellular and molecular analysis, FTY demonstrated the potential to be investigated as a novel neuroprotective strategy in conditions like MS and associated pathologies.

## Data Availability

Not applicable.

## Appendix A

**Table A1:** List of antibodies used in the study.

Antibody Used	Catalogue No.	Company	Dilution
Phospho-P38 MAP Kinase	4511S	Cell Signaling	1:1000
Total P38 MAP Kinase	9212S	Cell Signaling	1:1000
Phospho-Akt	4060S	Cell Signaling	1:1000
Total Akt	9272S	Cell Signaling	1:1000
Bcl-xL	2764S	Cell Signaling	1:1000
Cleaved caspase-3	9664S	Cell Signaling	1:1000
β-Actin	A1978-200UL	Sigma	1:10,000
Tuj1	MAB1195	R&D Systems	1:500
Neuron Specific Enolase	NSE	Aves Lab	1:500
Mitofusin-2	74792	Cell Signaling	1:1000
p-DRP-1	4494	Cell Signaling	1:1000
OPA-1	80471S	Cell Signaling	1:1000
